# Acute hyperglycaemia in cystic fibrosis pulmonary exacerbations

**DOI:** 10.1002/edm2.208

**Published:** 2020-11-30

**Authors:** Lina Merjaneh, Demet Toprak, Sharon McNamara, Laura Nay, Erin Sullivan, Margaret Rosenfeld

**Affiliations:** ^1^ Division of Endocrinology and Diabetes Seattle Children’s Hospital Seattle WA USA; ^2^ Department of Pediatrics University of Washington Seattle WA USA; ^3^ Division of Pulmonary and Sleep Medicine Seattle Children’s Hospital Seattle WA USA; ^4^ Children’s Core for Biomedical Statistics Center for Clinical and Translational Research Seattle Children’s Hospital Seattle WA USA

**Keywords:** continuous glucose monitoring, cystic fibrosis‐related diabetes, hyperglycaemia, insulin, oral glucose tolerance test

## Abstract

**Background:**

Hyperglycaemia may contribute to failure to recover from pulmonary exacerbations in cystic fibrosis (CF). We aimed to evaluate the prevalence and mechanism of hyperglycaemia during and post‐exacerbations.

**Methods:**

Nine paediatric CF patients, not on insulin, hospitalized for intravenous antibiotics, underwent an oral glucose tolerance test (OGTT) and continuous glucose monitoring (CGM) upon admission (visit 1) and an OGTT 2 weeks (visit 2) and 6 weeks to 12 months later when at stable baseline (visit 3). Insulin and glucose levels were measured before, 30, 60 and 120 min after glucose ingestion during OGTT. Hyperglycaemia on OGTT was defined according to the American Diabetes Association criteria as abnormal OGTT or consistent with diabetes. Hyperglycaemia on CGM was defined as CGM time above 140 mg/dL > 4.5%.

**Results:**

At visit 1, 8/9 patients had hyperglycaemia on both CGM and OGTT (2 diabetes and 6 abnormal OGTT). At visit 2, 5/8 had hyperglycaemia (all abnormal OGTT). At visit 3, (median (IQR) time since visit 1, 4.9 (3.8‐6.3) months), 5/7 had hyperglycaemia (2 diabetes and 3 abnormal OGTT). At visits 1, 2 and 3, respectively, mean (SD) 2‐hour OGTT glucose was 175.8 (42.3), 146.3 (31.9) and 176.9 (51.7) mg/dL. CGM time above 140 mg/dL at visit 1 was 25.3% (16.9). Insulin AUC decreased from visit 2 (median (IQR) 5449 (3321‐8123) mcIU‐min/mL) to visit 3 (3234 (2913‐3680) mcIU‐min/mL).

**Conclusion:**

Hyperglycaemia is prevalent during paediatric CF exacerbations; it appears to improve with exacerbation treatment but to worsen later in association with decreased insulin secretion.

## Graphical abstract

In this study, we evaluate the prevalence and mechanism of hyperglycaemia during and post‐cystic fibrosis exacerbations. We found that hyperglycaemia is prevalent during paediatric CF exacerbations using both oral glucose tolerance tests and continuous glucose monitoring. It appears to improve with exacerbation treatment but to worsen later in association with decreased insulin secretion.

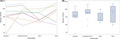

## 1 INTRODUCTION

Up to 25% of cystic fibrosis (CF) patients fail to recover to their baseline lung function after a pulmonary exacerbation,^1^ and recurrent pulmonary exacerbations are a risk factor for lung function decline and mortality.^2^, ^3^ Identifying factors that contribute to failure to recover is critical for designing better treatment for exacerbations. Hyperglycaemia is a potentially modifiable factor.

CF‐related diabetes (CFRD) development is associated with worse lung disease and accelerated lung function decline,^4^ and insulin therapy can improve that decline.^5^ In a retrospective study, poor glycemic control during exacerbations in children with CFRD was associated with poorer FEV1 recovery,^6^ suggesting that untreated hyperglycaemia can affect recovery from exacerbations. Two small studies in CF patients without diabetes reported contradicting results, with one study finding that patients exhibit diabetic glucose tolerance during exacerbations that returns to normal with recovery^7^ and the other finding little difference in glucose tolerance status during exacerbations compared to clinical stability.^8^ Furthermore, the mechanism underlying hyperglycaemia during exacerbations and the longer‐term changes in glucose tolerance following exacerbations have not been investigated.

We aimed to estimate the prevalence and severity of hyperglycaemia during pulmonary exacerbations in paediatric CF patients not on insulin, to determine whether hyperglycaemia improved at the end of exacerbation treatment and whether it remained stable after return to baseline respiratory status. We also aimed to evaluate the mechanism of hyperglycaemia by estimating insulin secretion and sensitivity during and postexacerbation.

## 2 METHODS

We recruited CF patients aged 10‐21 years hospitalized at Seattle Children's Hospital for management of a pulmonary exacerbation (ie new or increased respiratory symptoms or signs treated with intravenous (IV) antibiotics) between 2016 and 2019. We excluded patients if they were on insulin, pregnant or had a history of lung transplantation. A prior diagnosis of diabetes or abnormal oral glucose tolerance test (OGTT) was not an exclusion criterion.

Eligible patients participated in 3 study visits. The first, within 72 hours of admission, included an OGTT and 48 hours continuous glucose monitoring (CGM). The second visit occurred at 14 (± 2) days from visit 1 and the third as outpatient when at baseline health status (ie no treatment with systemic antibiotics in the previous 6 weeks and symptoms at baseline per participant report), 6 weeks to 12 months after visit 1; both included an OGTT. Historical data, including OGTT results during the preceding two years, were abstracted from the medical record.

With each OGTT, specimens were collected for blood glucose and insulin at baseline, 30, 60 and 120 minutes after ingestion of an oral glucose solution (1.75 g/kg up to 75 g). Glucose and insulin assays were performed at the Seattle Children's Hospital clinical laboratory. Glucose was measured by Vitros 4600 colorimetric method with an intra‐assay variability of 0.67%, inter‐assay variability of 0.8% and limit of quantification of 20 mg/dL. Insulin was measured by chemiluminescence on a Siemens Immulite 2000 XPI with intra‐assay variability of 5.5%, inter‐assay variability of 7.3% and limit of quantification of 2 mcIU/mL.

Hyperglycaemia on OGTT was defined according to the 2003 American Diabetes Association criteria^9^ and included diabetes (fasting glucose > 125 and/or 2‐hour glucose > 199), abnormal OGTT (fasting glucose between 100‐125 and/or 2‐hour glucose between 140‐199), or indeterminate glycemia (mid‐OGTT glucose > 200). Insulin sensitivity was calculated as 1/fasting insulin.^10^ Early‐phase insulin secretion was calculated as ∆insulin 0‐30min/∆glucose 0‐30min.^11^

CGM was performed using I‐pro2 (Medtronics) for 48 hours during the first 72 hours of admission. Hyperglycaemia on CGM was defined by glucose > 140 mg/dL for > 4.5% of time.^12^ Severity was evaluated by time spent over 140 and over 200 mg/dL.

### 2.1 Statistical analysis

Baseline clinical and demographic data are summarized descriptively. Total area under the curve (AUC) was calculated for OGTT glucose and insulin using the trapezoidal method for patients with data at each data point (fasting, 30, 60 and 120 minutes).

Box plots were plotted to show changes in 2‐hour glucose, 1‐hour glucose, glucose AUC and insulin AUC by visit. Line graphs were plotted to show changes in 2‐hour glucose and 1‐hour glucose by visit.

Within‐subject comparisons of glucose AUC and insulin AUC over time (baseline to visit 2, visit 2 to visit 3, and baseline to visit 3) were made using Wilcoxon signed rank tests. Given the small sample size, we limited these comparisons to two key metrics to minimize multiple comparisons; these results should be considered exploratory.

## 3 RESULTS

Nine patients completed visit 1. One patient was excluded after visit 1 as insulin was started for significant hyperglycaemia. Eight patients completed visit 2, and 7 completed visit 3. Table 1 shows the baseline characteristics of the participants.

**TABLE 1:** Baseline demographic and clinical characteristics of the study cohort (*N* = 9)

Characteristic	
Age (years)^a^	16.1 (2.1)
Gender (% female)	7 (77.8%)
Genotype (F508 del homozygous)	7 (77.8%)
Pancreatic insufficiency	9 (100%)
BMI Z‐score^a^	‐0.48 (1.46)
Systemic steroid use	1 (11.1%)
FEV1% at admission^b^	89 (82‐90)
Best FEV1% in the 12 months prior^b^	107 (82‐114)
Currently taking CFTR modulators	5 (55.6%)
Number of exacerbations in the 2 years prior	
0	1 (11.1%)
1	3 (33.3%)
2	2 (22.2%)
3 or more	3 (33.3%)
Most recent OGTT 2‐hour glucose (in 2 years prior) (mg/dL)^a^	158.3 (51.1)

^a^ Mean (SD).

^b^ Median (IQR).

Before enrolling in the study, 8/9 patients had hyperglycaemia on historical OGTT (2 diabetes and 6 abnormal OGTT) and 1 patient had a normal OGTT. At visit 1, 8/9 patients had hyperglycaemia (2 diabetes and 6 abnormal OGTT). At visit 2, 5/8 had hyperglycaemia (all abnormal OGTT, no diabetes). At visit 3, (median (IQR) time since visit 1, 4.9 (3.8‐6.3) months), 5/7 had hyperglycaemia (2 diabetes and 3 abnormal OGTT).

**FIGURE 1:**
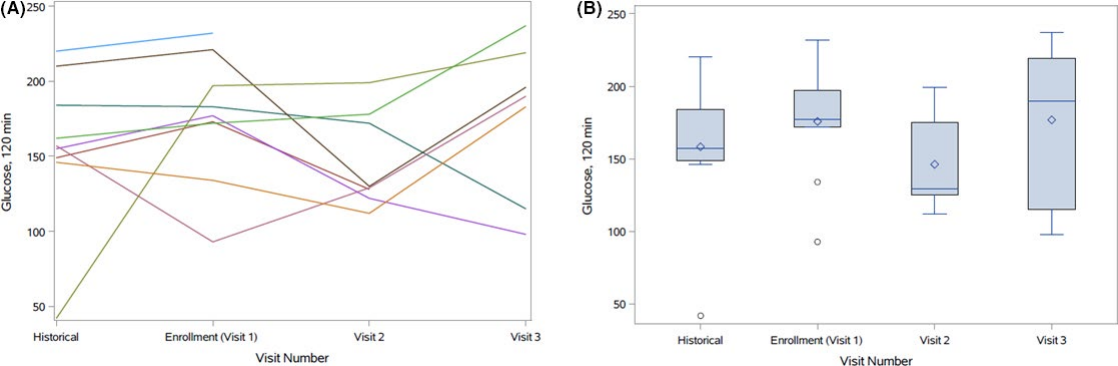
A and B, Distribution of 2‐hour glucose: prior to exacerbations, at baseline, 2 weeks and outpatient follow‐up

**FIGURE 2:**
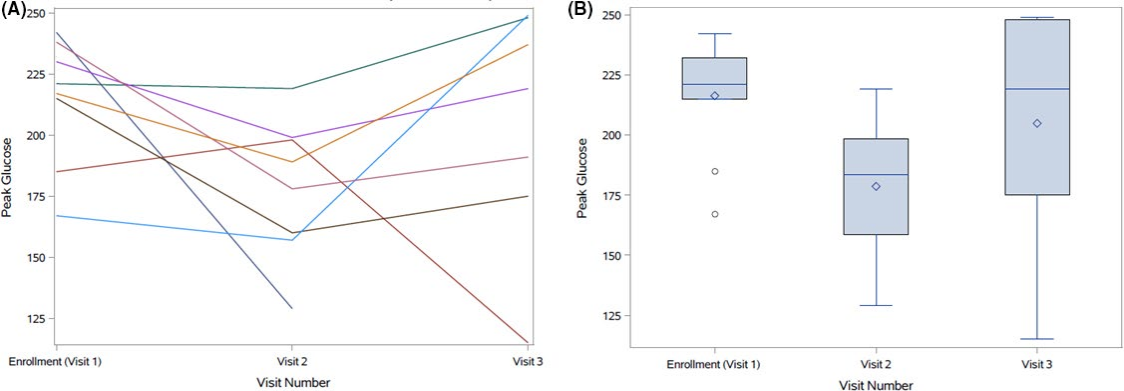
A and B, Distribution of 1‐hour glucose: at baseline, 2 weeks and outpatient follow‐up

**FIGURE 3:**
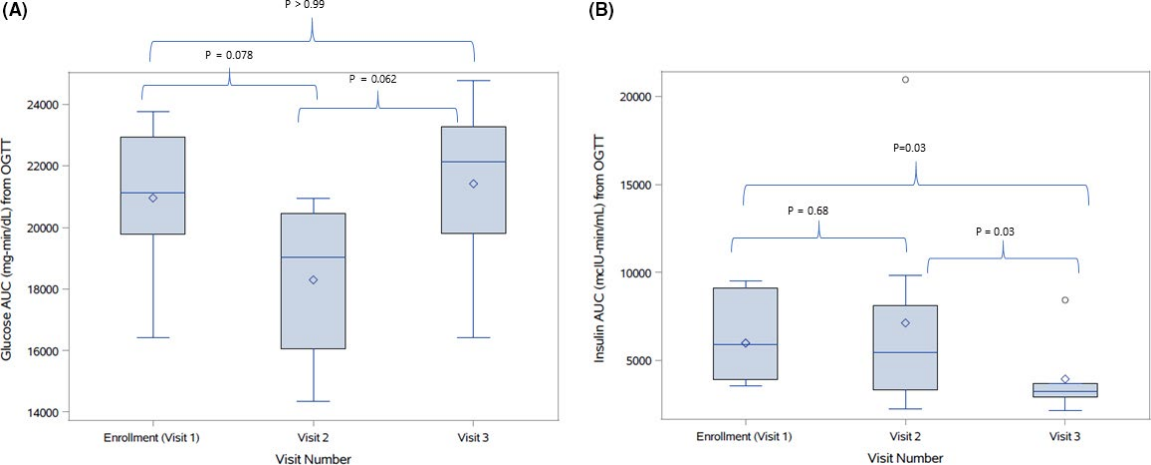
A, Distribution of glucose AUC: at baseline, 2 weeks and outpatient follow‐up. B, Distribution of insulin AUC: at baseline, 2 weeks and outpatient follow‐up

OGTT results are presented in Table 2 and Figures 1, 2, 3. The 1‐hour and 2‐hour glucose and the glucose AUC appeared lower at visit 2 compared to visit 1 but increased at visit 3. Insulin sensitivity appeared higher while insulin secretion and insulin AUC appeared lower at visit 3 compared to visits 1 and 2.

**TABLE 2:** Oral glucose tolerance test results

	Visit 1 *N* = 9^a^	Visit 2 *N* = 8	Visit 3 *N* = 7
Fasting glucose (mg/dL)^b^	91.6 (7.9)	91.9 (7.6)	89.1 (6.9)
1‐hour glucose (mg/dL)^b^	212.9 (28.6)	176.9 (27.8)	213.2 (30.5)
2‐hour glucose (mg/dL)^b^	175.8 (42.3)	146.3 (31.9)	176.9 (51.7)
Glucose AUC (mg‐min/dL)^b^, ^d^	20 957 (2388)	18 296 (2551)	21 423 (2956)
Insulin sensitivity mcIU/mL^‐1b^	0.12 (0.11 ‐ 0.20)	0.13 (0.05‐0.42)	0.32 (0.07‐0.50)
Insulin secretion^b^ (mcIU/mL)/( mg/dL)	0.39 (0.27‐0.56)	0.39 (0.34‐0.60)	0.23 (0.15)
Insulin AUC (mcIU‐min/mL)^c^, ^d^	5877 (3882‐9101)	5449 (3321‐8123)	3234 (2913‐3680)

^a^ Glucose and insulin AUC could not be calculated for two patients at visit 1 and one patient at visit 3 due to missing 30‐ and/or 60‐minute blood draws

^b^ Mean (SD)

^c^ Median (IQR)

^d^ Within‐subject comparison using Wilcoxon signed rank tests. Glucose AUC—visit 1 vs. visit 2: *P* = .078; visit 2 vs. visit 3: *P* = .062; visit 1 vs. visit 3: *P* > .99. Insulin AUC—visit 1 vs. visit 2: *P* = .68; visit 2 vs. visit 3: *P* = .03; visit 1 vs. visit 3: *P* = .03.

Mean (SD) CGM duration was 44.1 (11.6) hours, mean average glucose was 124.7 mg/dL (24.8), mean peak glucose was 243.9 mg/dL (65.9), and mean glucose SD was 41 mg/dL (23.4). Mean time spent > 140 mg/dL was 25.3% (16.9), and median (IQR) time spent > 200 mg/dL was 4% (1‐9). Hyperglycaemia was mostly postprandial in those who showed hyperglycaemia on CGM. CGM showed no hyperglycaemia in the patient with normal OGTT.

Between visits 2 and 3, BMI z‐scores declined by mean (SD) 0.19 (0.65), 2/7 patients were admitted for treatment of a pulmonary exacerbation, 6/7 patients received systemic antibiotics, and 1 patient was on chronic oral steroids (including visit 2 and 3) for allergic bronchopulmonary aspergillosis.

## 4 DISCUSSION

Our study shows that hyperglycaemia is highly prevalent during paediatric CF pulmonary exacerbations. Furthermore, glucose tolerance appeared to improve initially with exacerbation treatment but to worsen after the exacerbation, along with decreased insulin secretion. Previous studies of hyperglycaemia in CF pulmonary exacerbations followed patients only up to 6 weeks after the exacerbation.^7^, ^8^ By following patients longer, we were able to detect worsening glucose tolerance after initial improvement. Our study is also the first to compare OGTT and CGM results in the inpatient exacerbation setting. We found that CGM was feasible and reliable to detect hyperglycaemia compared to OGTT, suggesting that it may be useful in future studies evaluating hyperglycaemia during CF exacerbations.

Hyperglycaemia may impair recovery of lung health through both local and systemic effects. Increased airway glucose concentrations promote bacterial growth and impair innate immune function in animal models.^13^, ^14^ Systemic hyperglycaemia reduces neutrophil phagocytosis and chemokinesis.^15^ In critically ill non‐CF patients, treating hyperglycaemia results in significant decreases in morbidity and acquired infections during the hospitalization.^16^ The current CF Foundation guidelines recommend treating hyperglycaemia only if it persists after the first 48 hours of the exacerbation^17^ even in patients with a history of an abnormal OGTT. This approach may be missing an important window in which treating hyperglycaemia could potentially enhance and speed response to exacerbation treatment. Ultimately, our goal is to study whether insulin initiation upon admission in CF patients with hyperglycaemia can improve recovery from the exacerbation.

Hyperglycaemia appeared to improve after IV antibiotic treatment, which could be explained by reduced inflammation. Inflammation can exacerbate hyperglycaemia by increasing insulin resistance^18^ and decreasing insulin secretion.^19^ These measures did not appear to differ between visits 1 and 2, which could be due to the small sample size and the need for more rigorous methods of measurement.

Glucose tolerance appeared to worsen at visit 3, when patients had returned to their baseline respiratory status, in association with decreased insulin secretion. Glucose tolerance at visit 3 appeared worse than historical baseline prior to the exacerbation. Most of our patients had pulmonary exacerbations between visits 2 and 3 requiring oral or IV antibiotics. It is possible that exacerbation‐associated inflammation has an acute damaging effect on the beta cells, hastening a decline in insulin secretion ability. It is also possible that acute untreated hyperglycaemia during exacerbations has a toxic effect on beta cells, furthering the damage caused by inflammation. These results suggest that hyperglycaemia during exacerbations could be a risk factor for worsening glucose tolerance within the following year and treating it could potentially slow the decline in beta cell function.

Alternatively, worsening glucose tolerance could in turn be a risk factor for pulmonary exacerbations. Most of our patients had untreated abnormal or diabetic OGTTs prior to enrolling in the study that seemed to worsen at the end of the study. These patients also had frequent pulmonary exacerbations during the study. Whether treating abnormal OGTT can reduce exacerbation risk is not known, and prospective studies of insulin treatment are needed to address this question.

CGM has been used to diagnose hyperglycaemia in CF patients when well, and CGM‐guided insulin therapy has been shown to improve clinical outcomes in CFRD.^20^ In our study, CGM reliably identified hyperglycaemia and could potentially help guide insulin treatment during exacerbations.

The limitations of the study include the small sample size. Larger studies are needed to provide a more precise estimate of the prevalence of hyperglycaemia during CF exacerbations and its association with lung function and beta‐cell function decline. We included patients with abnormal or diabetic OGTT and on steroids in our study because our objective was to study the prevalence of hyperglycaemia during exacerbations in CF patients not on insulin, regardless of its aetiology. There was an expectedly large time window between visits 2 and 3 (6 weeks‐12 months), inviting undesired variability. Scheduling visit 3 at baseline health status was challenging as the participants had frequent exacerbations (6/7 patients received systemic antibiotics between visits 2 and 3). Lastly, OGTT results can be variable in CF patients over time^21^ and OGTT measures can be variable compared with intravenous testing.^22^ The insulin secretion measure that we used (∆I 0‐30/∆G 0‐30) is not thorough as it includes only two insulin measurements. However, this measure has been validated in CF patients and found to have a good correlation with measures obtained from IV glucose tolerance testing.^11^ The insulin sensitivity measure we used (1/fasting insulin) has also been validated in non‐CF patients and found to correlate well with insulin sensitivity measured via glucose clamps.^10^ The use of more rigorous methods of measurement such as IV glucose tolerance testing or glucose clamps is not feasible in sick, hospitalized patients.

In summary, we found that hyperglycaemia is prevalent during CF exacerbations, appears to improve with exacerbation treatment but to recur later in association with a decrease in insulin secretion. CGM reliably identified hyperglycaemia during CF exacerbations and could be used to guide its treatment. Future studies will evaluate the role of insulin treatment during pulmonary exacerbations.

## CONFLICT OF INTEREST

None.

## AUTHORS’ CONTRIBUTION

LM, D.T, SM and LN performed the research. LM, DM and MR designed the research study. ES analysed the data. LM and MR wrote the paper. All authors have read and approved the final manuscript.

This work was presented at the North American Cystic Fibrosis Conference in Nashville, TN, in October 2019.

## Data Availability

The data sets used and/or analysed during the current study are available from the corresponding author upon reasonable request.
